# Double Quantum Coherence ESR at Q-Band Enhances the Sensitivity of Distance Measurements at Submicromolar Concentrations

**DOI:** 10.1021/acs.jpclett.3c02372

**Published:** 2023-09-28

**Authors:** Alysia Mandato, Zikri Hasanbasri, Sunil Saxena

**Affiliations:** Department of Chemistry, University of Pittsburgh, Pittsburgh, Pennsylvania 15213, United States

## Abstract

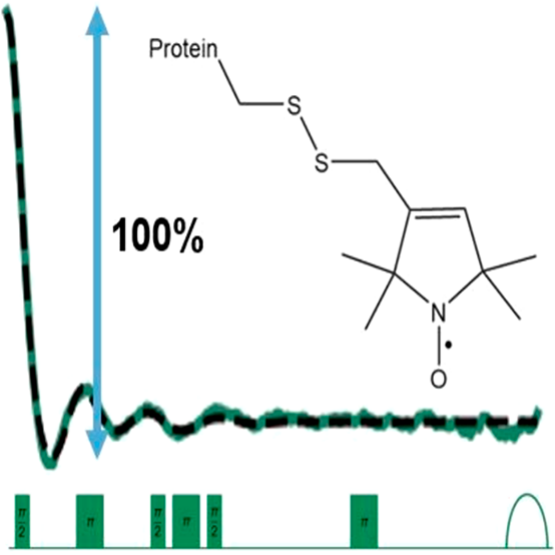

Recently, there have been remarkable improvements in pulsed ESR sensitivity, paving the way for broader applicability of ESR in the measurement of biological distance constraints, for instance, at physiological concentrations and in more complex systems. Nevertheless, submicromolar distance measurements with the commonly used nitroxide spin label take multiple days. Therefore, there remains a need for rapid and reliable methods of measuring distances between spins at nanomolar concentrations. In this work, we demonstrate the power of double quantum coherence (DQC) experiments at Q-band frequencies. With the help of short and intense pulses, we showcase DQC signals on nitroxide-labeled proteins with modulation depths close to 100%. We show that the deep dipolar modulations aid in the resolution of bimodal distance distributions. Finally, we establish that distance measurements with protein concentrations as low as 25 nM are feasible. This limit is approximately 4-fold lower than previously possible. We anticipate that nanomolar concentration measurements will lead to further advancements in the use of ESR, especially in cellular contexts.

Pulsed dipolar electron spin resonance (ESR) spectroscopy has recently emerged as an important tool in structural and mechanistic biophysics. In particular, methods such as double electron–electron resonance (DEER)^1−3^ or double quantum coherence (DQC),^4−6^ among others,^7−9^ provide 2–16 nm range distance constraints that relate to protein structure and flexibility. In these measurements, the magnetic dipolar interaction between a pair of site-specifically placed spin labels^10^ is measured in order to determine the distance between the unpaired electron spins. The nanometer-scale distance constraints are incisive probes of induced conformational changes,^11−16^ biomolecular interactions,^17−19^ and ligand binding site characterization.^20−22^ Pulsed dipolar ESR measurements are also an attractive avenue to measure structural constraints in cellular environments^23−26^ and in large membrane proteins.^27−30^

However, ESR distance measurements are typically performed with protein concentrations at the micromolar level. An emerging need in the pulsed ESR field is to increase the sensitivity of the techniques. The ability to investigate proteins at submicromolar concentrations is beneficial in cases where protein overexpression and purification are difficult or where protein solubility is low. Low concentration sensitivity is also important to the measurement of submicromolar binding affinities and equilibrium properties.^31,32^ Aside from low concentration measurements, high sensitivity also aids in the interpretation of ESR distance distributions with multiple components.^3^

For these reasons, there has been much work to improve and calibrate the sensitivity of many pulsed dipolar ESR methods. Solvent and protein deuteration have prolonged phase memory times of samples,^33−35^ cryogenic amplifiers have decreased measurement times,^36−38^ and spectrometers with low-noise microwave amplifiers have resulted in relaxation measurements of only 10^7^ spins.^39^ These achievements are paving the way for a broader applicability of ESR in the measurement of biological distance constraints, for instance, at physiological concentrations and in more complex systems. Recent work has shown the capability of pulsed dipolar ESR to reach submicromolar protein concentration measurements *in vitro*(32,40−43) and in cell.^44^ Current techniques appear to work with protein concentrations as low as 45–200 nM^40,42,44^ depending on the spin label, but these measurements often require a few days of collection time at these low concentrations. Therefore, there remains a need for rapid and reliable methods of measuring distance distributions between spins at submicromolar concentrations.

In this work, we explore the use of DQC at Q-band to acquire distance constraints on protein systems with multimodal distance components or at submicromolar concentrations. DQC was pioneered and developed at X-, Ku, and Q-band frequencies on systems labeled with nitroxide,^45−50^ Cu^2+^,^51−54^ and trityls.^42^ Previous Q-band DQC work has only been reported on trityl-labeled proteins,^42,43,55−58^ as nitroxide-based Q-band DQC has inherent challenges. The nitroxide spectrum at the Q-band frequency is broad enough that it is difficult to efficiently excite the double quantum coherence with pulse lengths that are available on most spectrometers. In the following, we achieve efficient excitation of the double quantum coherence with a recently available, high power, loop-gap resonator (Bridge12 Technologies, Inc.).^59^ We explore the advantages of Q-band nitroxide DQC for interspin distance determination. We also highlight the ability of the method to efficiently measure distances at the lowest protein concentrations reported by pulsed dipolar ESR spectroscopy thus far.

The protein we used in this work was the B1 immunoglobulin binding domain of protein G (GB1). We expressed and purified the E15C/K28C mutant of GB1, which has been previously studied by ESR distance measurements.^60,61^ The protein was labeled with (1-oxyl-2,2,5,5-tetramethylpyrroline-3-methyl)methanethiosulfonate spin label (MTSSL) to yield the R1 side chain (Figure 1A). The labeling efficiency was 100% as determined by continuous-wave ESR (CW-ESR) (Figure S1 and Table S1 in the Supporting Information).

**Figure 1:**
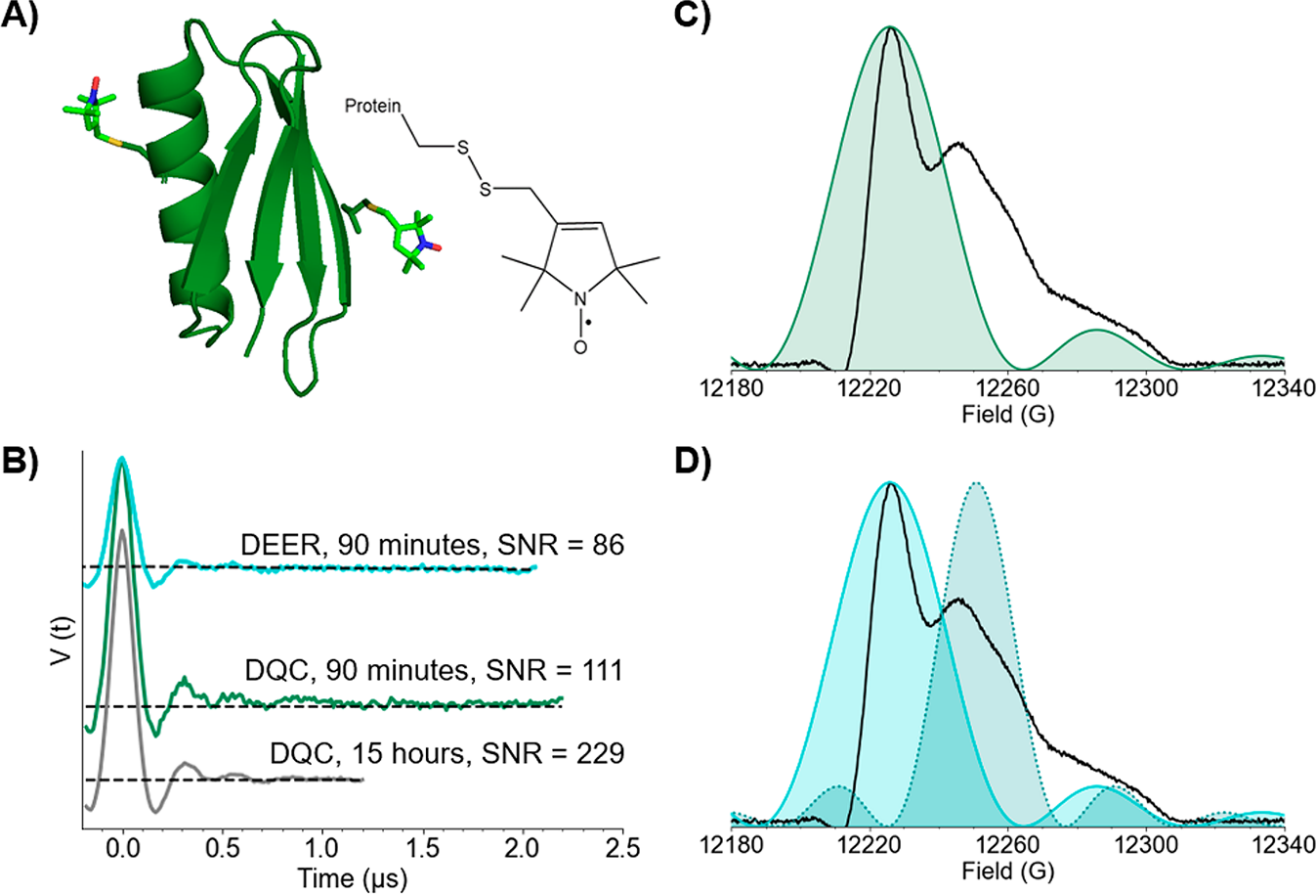
(A) Model of GB1 (PDB: 2QMT) with 15R1/28R1 positions of the nitroxide spin label MTSSL shown in stick representation (left) and the chemical structure of the R1 nitroxide spin label (right). (B) DEER (blue, top) and DQC (green/gray, middle/bottom) time traces collected at 50 K using 10 μM protein. Dashed black lines represent the intermolecular background fits by DeerAnalysis.^62^ (C) Echo-detected field swept spectrum of 15R1/28R1 GB1 (black). The green area represents the theoretical excitation profile of the 8 ns rectangular pulse used for DQC. (D) Theoretical excitation profiles of a 12 ns rectangular observer pulse (dotted blue) and an 8 ns rectangular pump pulse (solid blue) used for DEER.

We then collected Q-band DEER and DQC time domain signals using 10 μM of the protein. DEER is the most commonly used and well-established pulsed dipolar technique, and therefore we used it as a baseline comparison for our Q-band DQC experiments. Figure 1B compares the Q-band DEER and DQC experiments performed on 15R1/28R1 GB1. The DEER time trace is shown as the blue line, while the DQC time trace is shown as the green line. We also included a DQC time trace with a longer collection time, shown as the gray line.

Using 8 ns π pulses, the DQC signal was acquired at a single frequency at the center of the resonator bandwidth and the maximum of the echo-detected field-swept spectrum (Figure 1C). Q-band DQC experimental parameters are provided in Table S2, and optimization experiments are provided in Figures S2–S4. Remarkably, we observed a modulation depth of ca. 100% at this position. The 100% modulation depth was possible due to the combination of the short pulses with the enhanced *B*_1_ field strength of the loop-gap resonator and 300 W traveling-wave tube (TWT) amplifier used in this work. These results are notable because the typical modulation depths for alternative PDS techniques, like DEER and RIDME, are less than 50%.^3,40^ On the other hand, an out-of-phase DEER approach is available which provides ca. 100% modulation depth, but this comes with a four times reduction in sensitivity.^63^ For the DEER experiment, we used an 8 ns pump π pulse and a 12 ns observer π pulse, as shown in Figure 1D. The 43% modulation depth was within expectations given the enhanced excitation bandwidth of the 8 ns pulse. Note that Q-band nitroxide DEER typically results in ca. 35% modulation depths using 12–16 ns pump π pulses.^3,64^ The experimental parameters for DEER are provided in Table S3.

Given the differences in modulation depth between DEER and DQC, we calculated the signal-to-noise ratio (SNR) for each time trace by the equation SNR = λ/σ_noise_, where λ is the modulation depth and σ_noise_ is the root-mean-square deviation of the noise.^3,65^ The data in Figure 1B were collected with high SNR of 86 for DEER and 111 for DQC in 90 min each. Figure 2A shows the background-subtracted time traces for DEER and DQC. Note that the noise in DQC appears to be larger because of the increased modulation depth. However, the noise in the DQC data is similar to DEER, as is evident when the DEER data are scaled to the same modulation depth as DQC (cf. Figure S5). It is also instructive to compare the DEER conducted in this work with previous work on the same protein with a 150 W amplifier and conventional Q-band resonator.^40^ Despite a lower resonator active volume and protein concentration, we achieve a similar SNR by the use of shorter pulses and a 300 W amplifier (cf. Table S4).

**Figure 2:**
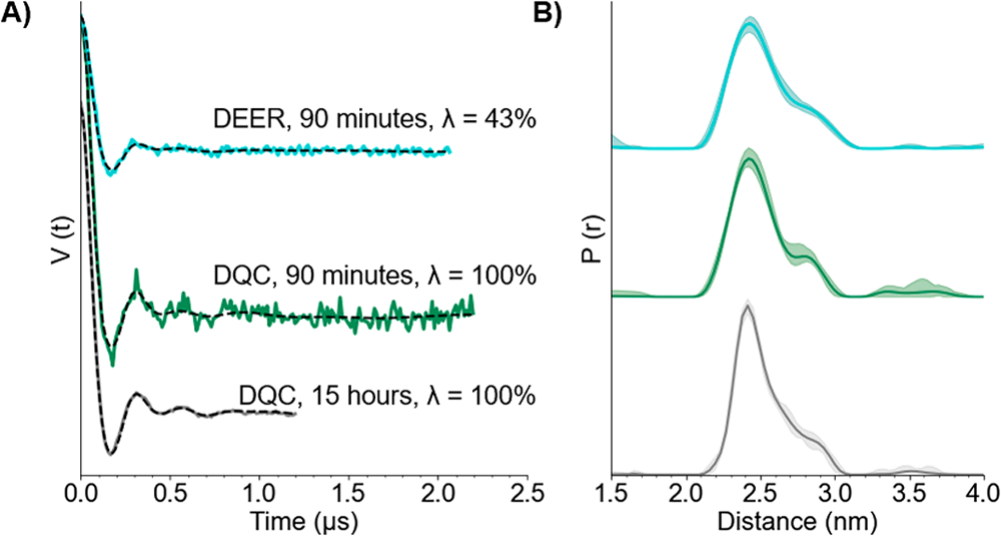
(A) Background subtracted DEER (blue, top) and DQC (green/gray, middle/bottom) time traces of 10 μM 15R1/28R1 GB1. Dotted black lines indicate the fit of the data by DeerAnalysis. (B) Distance distributions obtained from the DEER and DQC signals with shading to represent error. Distances distributions and validations were provided by DeerAnalysis.

Figure 2B shows the distance distributions processed by DeerAnalysis.^62^ The distance distributions match reasonably well in both the shape and the most probable distance of 2.4 nm. The uncertainty in the distributions computed as the 95% confidence interval is shown as shaded regions in the distributions. Note that DeerAnalysis uses a two-step procedure that estimates the intermolecular contribution and removes it from the signal by division. This process can be difficult for data with large modulation depths that have low intermolecular background signals and can lead to an overestimation of uncertainty. Indeed, analysis of the data using DEERNet^66^ leads to a similar uncertainty for both distance distributions from DQC and DEER (cf. Figure S6). Overall, we hypothesized that the deep modulation depth from the DQC experiment can be utilized for both resolving bimodal distances and analyzing distance distributions at low concentrations.

We generated and spin-labeled an additional V21C/G38C GB1 mutant with a longer interspin distance. CW-ESR data showed a labeling efficiency of 87% for this mutant (cf. Figure S1). We then collected the DEER and DQC signals for 10 μM 21R1/38R1 GB1, as shown in Figure 3B as pink and purple lines, respectively. It is clear that the modulations in the dipolar signals are lower in frequency than those in the 15R1/28R1 GB1 mutant, shown in Figure 2A. The 36% and 82% modulation depths of the DEER and DQC experiments, respectively, are slightly lower than those of the 15R1/28R1 experiments. We attribute the decrease in modulation depth due to the lower labeling efficiency of MTSSL to this mutant. Despite the decrease in modulation depth, we were able to analyze the time traces from both DEER and DQC. From the background-subtracted data, the distance distributions were acquired, as shown in Figure 3D. The most probable distances are in reasonable agreement at 3.6 nm.

**Figure 3:**
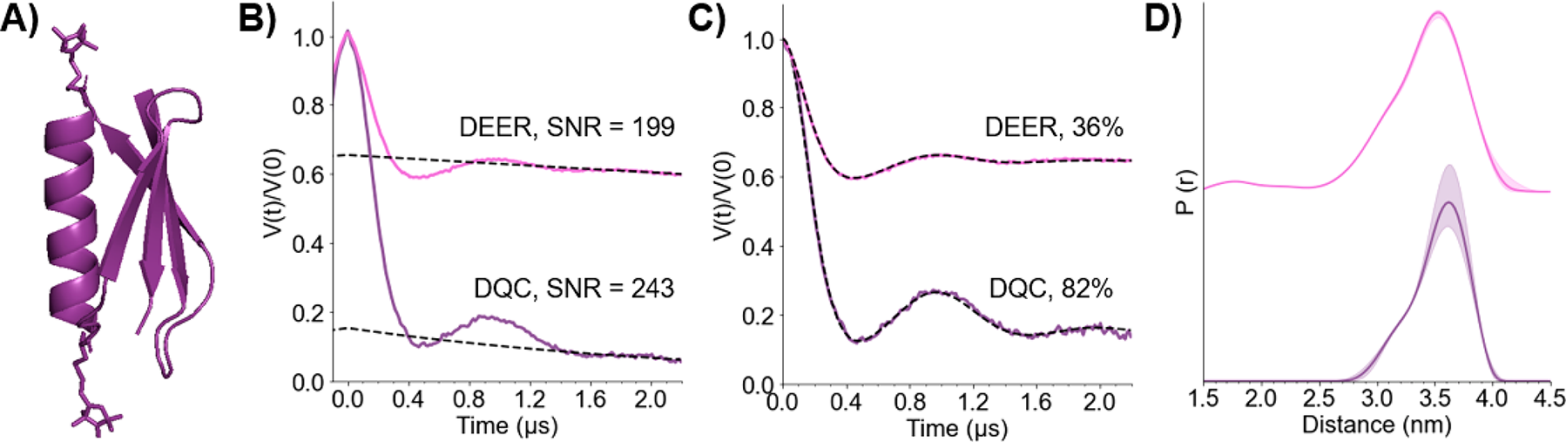
(A) Model of GB1 (PDB: 2QMT) with 21R1/38R1 positions of the nitroxide spin label MTSSL in stick representation. (B) DEER (pink, top) and DQC (purple, bottom) time traces collected at 50 K using 10 μM protein. Dashed black lines represent the intermolecular background function of the dipolar signal. (C) Background-subtracted time traces. Dashed black lines indicate the fits of the data by DeerAnalysis. (D) Distance distributions and corresponding validations provided by DeerAnalysis.

Next, we performed DQC on 10 μM protein concentration mixtures of the 15R1/28R1 and 21R1/38R1 GB1 mutants in different ratios to generate bimodal distance distributions. The background-subtracted data are shown in Figure 4A, and the primary DQC time traces are shown in Figure S7. In most of the time traces, the two frequency components in the background-subtracted time domain signals are easily visible. For example, in looking at the 50:50 data in Figure 4A (third from the bottom), there is a high-frequency component present at around 0.2 μs and a longer-frequency component visible at 1.0 μs. Figure 4B shows the extracted distance distributions from each time trace acquired by DeerAnalysis. For these analyses, an inclusion of additional white noise was necessary for validation (cf. details in the Supporting Information). Analysis of these data with alternative programs is provided in Figure S8. As the concentration of one mutant decreases, the population of the mutant in the distribution clearly decreases, and vice versa. To validate these results, the experimental distance distributions, shown as solid lines, were compared to those generated by addition of the two component distributions, shown as dotted lines. The distributions were normalized to the areas under the curves between 1.8 and 4.4 nm. Most of the dotted line components are within the shaded error of the experimental distributions, indicating good agreement. However, for the 87.5:12.5 mixture (bottom), we observed a longer distance component that is completely within the error of the distribution. This finding is due to the lower labeling efficiency of the 21R1/38R1 GB1 mutant. We anticipated that the 3.6 nm experimental distance would be undersampled in the mixture populations, which is clearly visible with this 87.5:12.5 mutant. Overall, the large modulation depth of the Q-band DQC experiment allows for clear and efficient analysis of bimodal dipolar signals.

**Figure 4:**
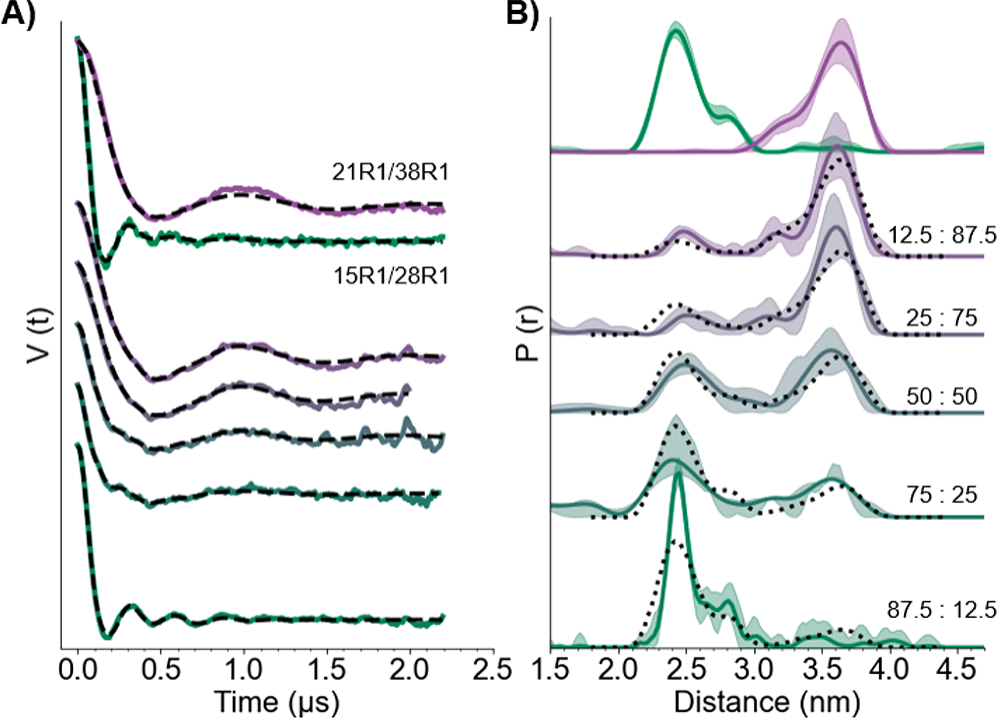
(A) Background subtracted DQC time traces for mixtures of 15R1/28R1 and 21R1/38R1 GB1. The top time traces are the DQC signals from the individual constructs. The next time trace has the lowest ratio of 15R1/28R1 to 21R1/38R1 (12.5 μM:87.5 μM), and the ratio increases from to top to bottom. (B) Corresponding distance distributions of GB1 mixtures predicted by DeerAnalysis. The dotted black lines are from the addition of distributions from each construct in the corresponding mutant ratios. The distributions were normalized to the areas under the curves between 1.8 and 4.4 nm.

Next, the large modulation depth and short collection time of the DQC experiment prompted us to explore the concentration sensitivity. Figure 5A shows the 12 h DQC and DEER time traces obtained on a sample containing 50 nM 15R1/28R1 GB1. The frequency modulations in the DQC are more visible, due to the large 100% modulation depth compared to 20% for DEER. The lower modulation depth of the DEER data for the 50 nM sample compared to the 10 μM sample (Figure 5B vs Figure 2A) is unclear, but such discrepancies at lower concentrations have been reported previously.^44^ Distance distributions were obtained using DeerAnalysis (Figure 5C); however, the SNR of the 50 nM DEER was not high enough to obtain a reliable distance distribution using Tikhonov regularization. Further details are available in Figure S9. The distribution of the 50 nM 15R1/28R1 GB1 DQC matches quite well with that of the 10 μM sample (Figure 2B). Because we were able to acquire the 50 nM signal relatively easily, we reduced the protein concentration even lower to 25 nM. As shown in Figure 5, we collected a DQC time trace of the 25 nM protein sample in 40 h. The modulations are relatively clear, even at this low concentration, due to the deep modulation depth. The most probable distance is consistent with those of both the 10 μM and 50 nM samples. We then attempted to measure the DQC of a 10 nM protein sample, but the SNR was low after 36 h. Therefore, distance measurements below the 25 nM protein concentration are currently prohibitive with respect to time (Figure S10).

**Figure 5:**
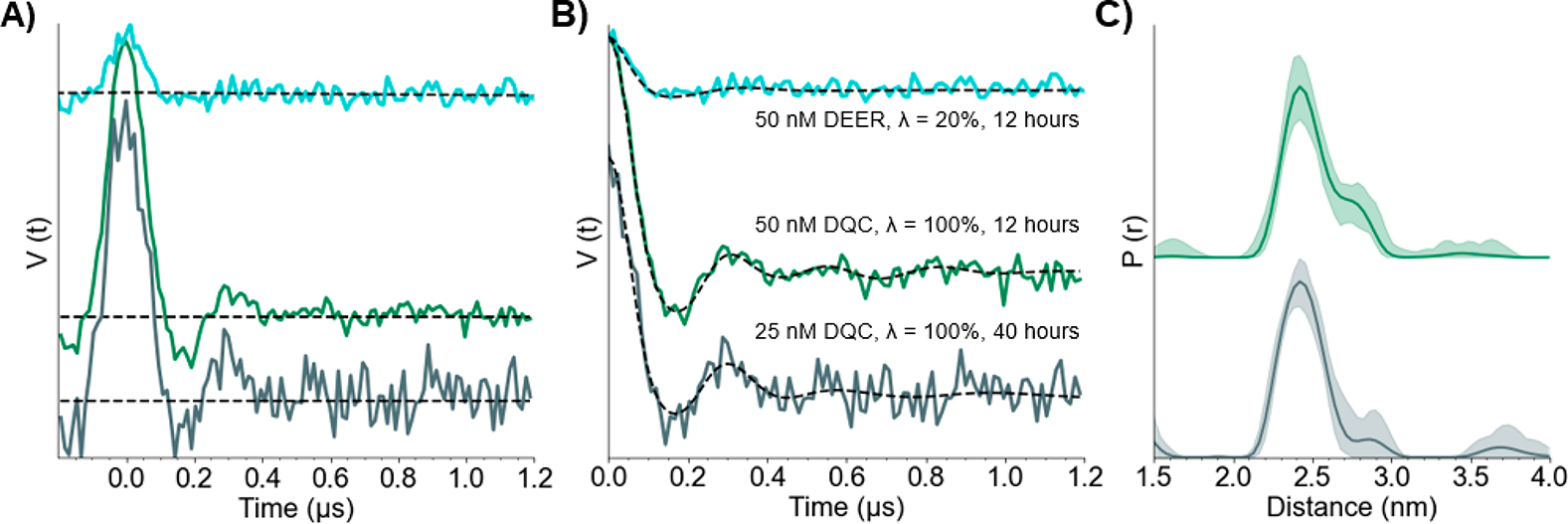
(A) DEER (blue, top) and DQC time traces of 50 nM 15R1/28R1 GB1 (green, middle) and 25 nM 15R1/28R1 GB1 (gray, bottom). (B) Background subtracted time traces. (C) Distance distributions obtained from the DQC signals with shading to represent error obtained by DeerAnalysis.

More importantly, we were able to measure an interspin distance distribution at only 25 nM protein concentration in 40 h using Q-band nitroxide DQC. Currently, measuring distances of submicromolar protein samples by pulsed ESR requires a few days to produce time traces with sufficient SNR,^40,42,44^ and the lowest nitroxide-based measurement is at 100 nM protein in 48 h.^40^ Comparatively, our 50 nM data is 2 times lower in protein concentration in about one-fourth of the collection time. We were also able to obtain a distance distribution of a 4 times lower protein concentration in about the same amount of time as the previous report for 100 nM protein. It is also important to note that in this work we used a short dipolar evolution time. For longer distances, the phase memory times of the echo can be limiting. While these can be combated by protein deuteration,^33,34^ a five-pulse DEER sequence is available that can partially reduce the effects of electron–nuclear interactions.^67^ It will be interesting to explore similar schemes for DQC.

In summary, we show that DQC using nitroxide-labeled protein is possible at Q-band frequencies. Until now, Q-band DQC has been achieved with trityl-based labels only because the spectrum is approximately 10 times narrower than nitroxide.^65^ The modulation depths in this work are 100%, allowing for exploration of the bimodal distance distribution analysis. With this technique, we were able to measure a 2.4 nm distance on 25 nM protein in only 40 h. The sensitive method demonstrated here provides an opportunity to improve future distance measurements that are multimodal or limited by concentration.

## Supporting Information Available

Protein expression and purification protocols, labeling efficiency CW-ESR spectra, DQC and DEER experimental parameters, DQC optimization experiments, pulsed dipolar analysis methods and details, and raw DQC time traces (PDF)
