# Supplementary material for: Double Quantum Coherence ESR at Q-Band Enhances the Sensitivity of Distance Measurements at Submicromolar Concentrations
Source: J Phys Chem Lett. 2023 Sep 28;14(40):8909–15. doi: 10.1021/acs.jpclett.3c02372 (PMC10577775; doi:10.1021/acs.jpclett.3c02372)
Supplement: Supplementary file 1 — jz3c02372_si_001.pdf [file jz3c02372_si_001.pdf]

SUPPORTING INFORMATION

Double Quantum Coherence ESR at Q-band Enhances the Sensitivity of Distance Measurements at Sub-Micromolar Concentrations

*Alysia Mandato, Zikri Hasanbasri, and Sunil Saxena**

Department of Chemistry, University of Pittsburgh, Pittsburgh, PA 15213, United States

Table of Contents

Protein expression and purification	S3
ESR sample preparation	S3
ESR experimental parameters	S3-S4
Figure S1. X-band CW-ESR spectra	S5
Table S1. Labeling efficiencies of R1-labeled GB1 mutants	S5
Table S2. DQC experimental parameters	S6
Figure S2. Q-band DQC optimization of pulse separations	S7
Figure S3. Q-band DQC optimization of pulse lengths	S8
Figure S4. Q-band DQC optimization of field and frequency	S8
Table S3. DEER experimental parameters	S9
Figure S5. Comparison of SNR in 10 μM 15R1/28R1	S10
Analysis of pulsed dipolar ESR time traces	S10
Figure S6. Comparison of analysis methods of DQC and DEER	S11
Figure S7. Raw and background-subtracted DQC time traces for mixtures	S12
Figure S8. Comparison of analysis methods of 15R1/28R1 GB1 DQC	S13
Figure S9. Estimation of Tikhonov regularization parameter for low concentration measurements	S14
Figure S10. 10 nM GB1 DQC	S14
Table S4. Comparison of DEER experiments performed with different equipment and parameters	S15
Figure S11. Estimation of Tikhonov regularization parameter for mixture measurements	S16
Figure S12. GB1 mutant purification	S16
References	S17

EXPERIMENTAL

Protein preparation

The protein used in this work was the B1 immunoglobulin binding domain of protein G (GB1). Mutant plasmids were purchased from SynBio Technologies. GB1 mutants were expressed and purified as previously described[1,2] and stored in phosphate buffered saline (PBS) at pH 7.4. Purification was confirmed via SDS-PAGE (Figure S1).

ESR Sample preparation

The purified protein incubated with 5 mM dithiothreitol (DTT) for two hours at room temperature to reduce disulfide bonds. The reducing agent was subsequently removed using five 5 mL HiTrap desalting columns (Cytiva Life Sciences). The reduced protein fraction was collected directly into a solution of 10-fold molar excess of (1-oxyl-2,2,5,5-tetramethylpyrroline-3-methyl) methanethiosulfonate (MTSSL). The solution was incubated overnight at 4 °C with constant agitation. Any unreacted spin label was removed by running the solution through the same desalting columns with PBS, pH 7.4. The spin-labeled protein fraction was concentrated through centrifugal filter units with a 3 kDa MWCO (MilliporeSigma). During concentration, the protein was buffer-exchanged into 50 mM 3-(N-morpholino)propanesulfonic acid (MOPS), pH 7.4 and 100 mM NaCl in deuterated water. The final protein concentration was quantified by UV-Vis measurement using the NanoDrop 2000 spectrophotometer (Thermo Scientific) using the GB1 extinction coefficient of 9970 L $mol^{-1}cm^{-1}$. The labeling efficiency was quantified to be 87% and 100% for each mutant (Table S1) according to quantitative ESR spin-counting experiments on a Bruker spectrometer (Figure S2).

For continuous wave (CW)-ESR experiments, 20 μL of solution were prepared and drawn into Pyrex capillary tubes (0.8 mm I.D. x 1 mm O.D. x 40 mm length). Capillary tubes were sealed and placed into ESR sample tubes (3 mm I.D. x 4 mm O.D. x 250 mm length). ESR samples for pulsed experiments were prepared to a final volume of 15 μL in the same MOPS/NaCl buffer in 40% v/v deuterated glycerol. For pulsed ESR experiments, the solutions were inserted into clear fused quartz capillary tubes (1.1 mm I.D. x 1.6 mm O.D. x 100 mm length, one end sealed) and were flash-frozen in liquid methylacetylene-propadiene propane (MAPP) gas.[3]

EPR measurements

Room-temperature CW-ESR experiments were performed on the Bruker ElexSys E680 FT/CW X-band (~9.8 GHz) spectrometer with a Bruker ER4122 SHQE-W1 high-resolution resonator. CW-ESR experiments were run at a center field of 3512 G with a sweep width of 150 G, modulation amplitude of 1 G, and modulation frequency of 100 kHz for a total of 1024 data points using a conversion time of 20.48 ms. CW-ESR spectra are provided in Figure S2.

Dead-time free 4-pulse DEER[4] experiments at Q-band frequencies (~34 GHz) were performed at 50 K using a Bruker ElexSys E580 FT/CW spectrometer with the Bridge 12 Technologies QLP Q-band resonator and a 300 W TWT amplifier. The temperature of the experiment was maintained using an Oxford CF935 dynamic continuous-flow cryostat attached to an Oxford LLT 650 low-loss transfer tube and an Oxford ITC503 temperature controller. The following DEER pulse sequence was used: $(\pi/2)_{\omega A} - \tau_1 - \pi_{\omega A} - (\tau_1 + T) - \pi_{\omega B} - (\tau_2 - T) - \pi_{\omega A} - \tau_2$ – echo. 16-step phase cycling was used in DEER to remove unwanted signals.[4] For the 10 μM samples, the observer pulse lengths, $(\pi/2)_{\omega A}$ and $\pi_{\omega A}$, were 6 and 12 ns, respectively. The pump pulse, $\pi_{\omega B}$, was 8 ns. For the 50 nM samples, the observer pulse lengths, $(\pi/2)_{\omega A}$ and $\pi_{\omega A}$, were 4 and 8 ns, respectively. The pump pulse, $\pi_{\omega B}$, was 12 ns. All pulses used were rectangular pulses. DEER parameters are provided in Table S3.

6-pulse DQC[5,6] was performed using the same spectrometer and resonator as DEER. A 256-step phase cycling program (64 steps combined with CYCLOPS) was used.[7] The following DQC pulse sequence was used: $(\pi/2)–t_p–\pi–t_p–(\pi/2)–t_1–\pi–t_1–(\pi/2)–(t_m–t_p)–\pi–(t_m–t_p)$–echo. The π/2 and π pulses were 4 and 8 ns, respectively. The initial delays t_p and t_1 were 200 and 146 ns, respectively, and the delay t_m-t_p was 1200 ns for a 2200 ns acquisition time. The time t_p was stepped out by 6 ns for a total of 200 points. All measurements were obtained with 20 shots per data point and a 3 ms shot repetition time. DQC parameters are provided in Table S2.

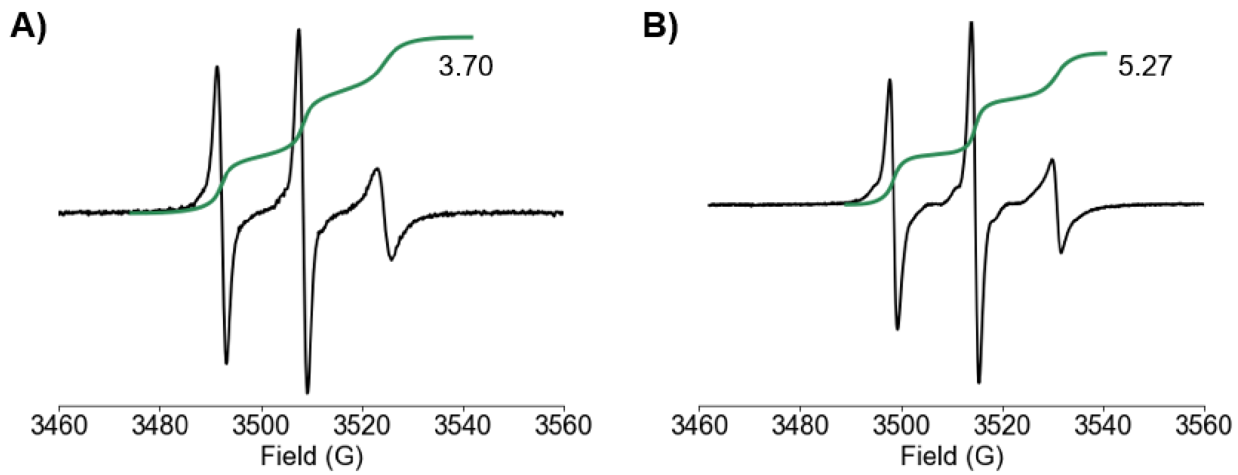

Figure S1. X-band CW-ESR spectra of the R1 GB1 mutants. (A) E15R1/K28R1 and (B) V21R1/G28R1. The green lines are the double integrals of the spectra, and the values are provided.

Table S1. Labeling efficiencies of R1-labeled GB1 mutants.

**Mutant**	**Protein conc (uM)**	**Spin conc. (uM)**	**Labeling efficiency (%)**
E15R1/K28R1	50	103	100%
V21R1/G28R1	50	87	87%

Table S2. DQC acquisition parameters, modulation depths, and SNR.

	15R1/28R1				21R1/38R1	Mixtures
GB1 Concentration (μM)	10	0.05	0.025	0.01	10	10
π/2 (ns)	4	4	4	4	4	4
π (ns)	8	8	8	8	8	8
t_p (ns)	200	200	200	200	200	200
$(t_m - t_p)$ (ns)	2400	1400	1400	1400	2400	2400
t_1 (ns)	146	146	146	146	156	156
d30 (ns)	6	6	6	6	6	6
d31, J	2, 64	2, 64	2, 64	2, 64	2, 64	2, 64
a	13	13	13	13	13	13
Shots per point	20	20	20	20	20	20
Scans	1	1	1	1	1	1
SRT (ms)	3	4	4	4	1	1
Attenuation (dB)	3	3	3	3	3	3
Points	200	117	117	117	209	209
Phase cycling	256-steps	256-steps	256-steps	256-steps	256-steps	256-steps
Modulation depth (%)	100	100	100	100	82	Varies, see Figure S8.
SNR (λ/σ_N)	111	37	15	n/a	243	Varies, see Figure S8.
Run time (hours)	1.5	12	40	36	1.67	Varies, see Figure S8.

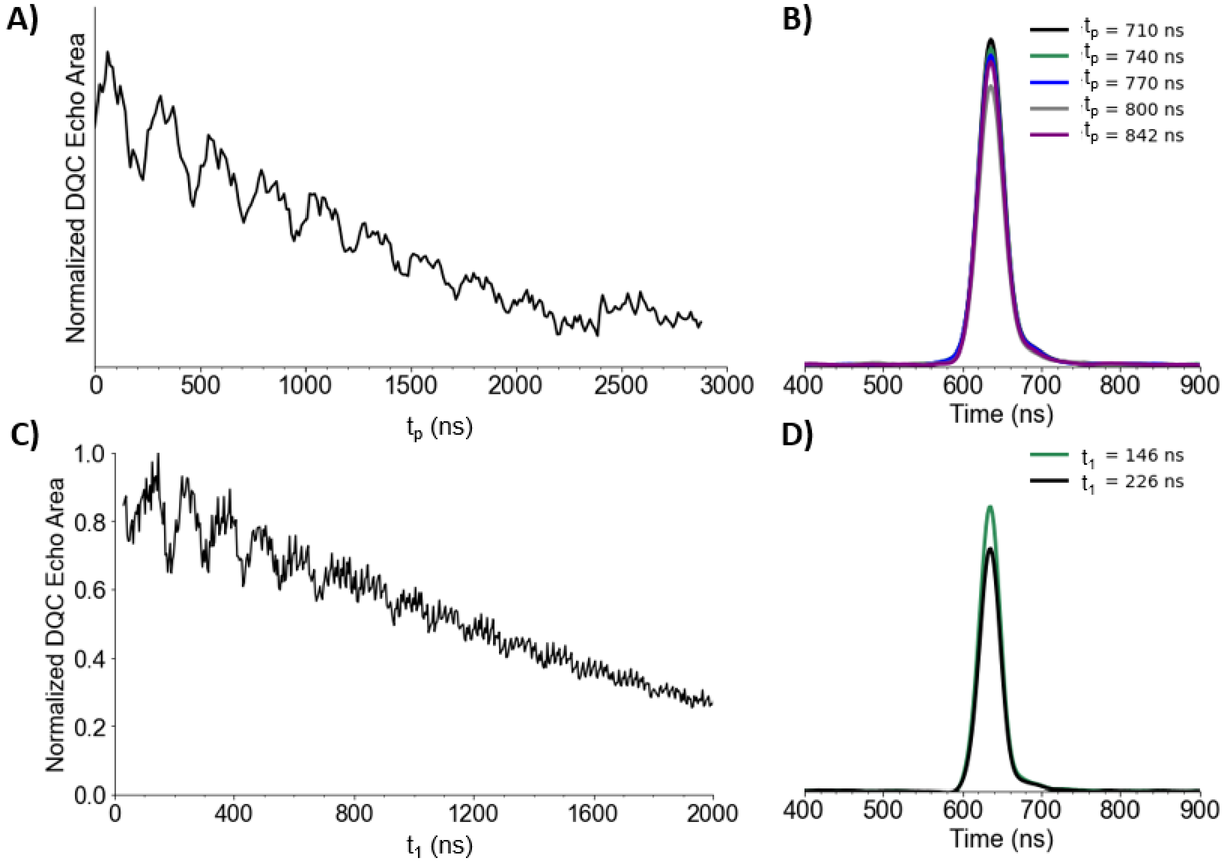

Figure S2. Optimization of DQC pulse separations. (A) The DQC echo area was measured as a function of pulse separation t_p. The acquisition time required for the GB1 distance needed a t_p of at least 700 ns, so we focused on the features in the plot around 700-900 ns. (B) DQC echoes for various t_p pulse separations. Since all values were similar in intensity, we went with the even 800 ns. (C) DQC echo area was measured as a function of pulse separation t_1. (D) The DQC echo was plotted using the peaks on the t_1 plot at 146 ns and 226 ns. 146 ns was the optimal pulse separation.

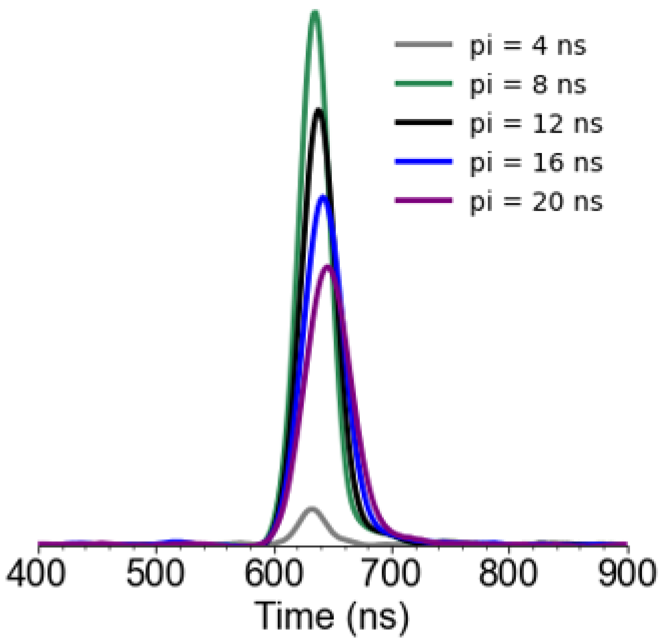

Figure S3. DQC optimization of pulse lengths. Based on DQC echo intensity and area, the best π pulse length for the experiment was 8 ns. Longer pulse lengths resulted in smaller echoes. This can be explained by the idea that longer pulses have shorter excitation bandwidths. However, the 4 ns pulse length resulted in a very small DQC echo. We attribute this discrepancy to the inability of the spectrometer to create a true 4 ns pulse based on the rise time of the pulses.

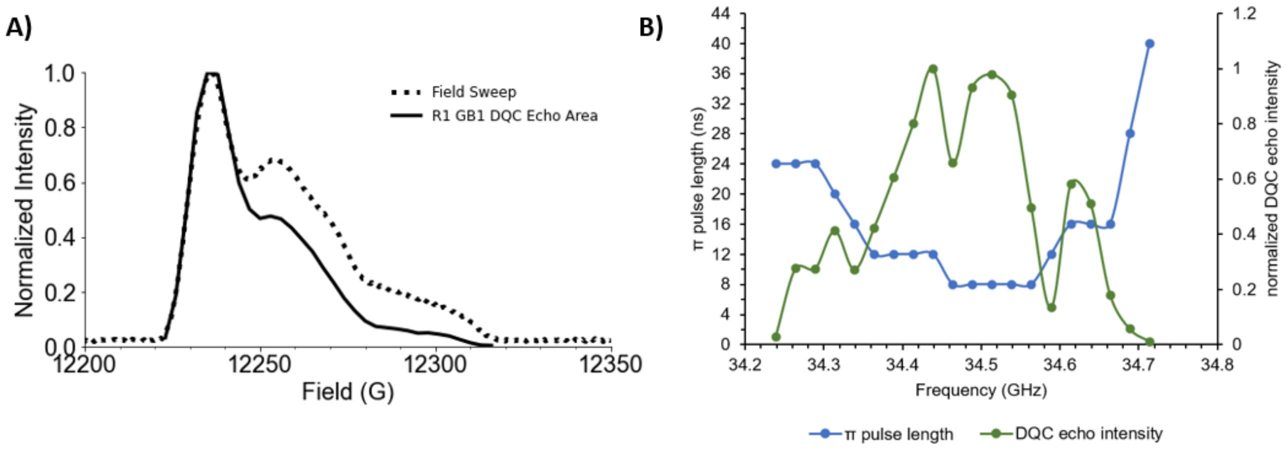

Figure S4. DQC optimization of field and frequency. (A) Q-band nitroxide echo-detected field swept spectrum (dotted) and DQC echo area as a function of field (solid). We find that the DQC echo area is the greatest at the maximum of the field sweep, so all DQC experiments were performed there. (B) Pulse length (blue) and DQC echo area (green) as functions of microwave frequency. The blue line represents the bandwidth of the Bridge12 Technologies QLP resonator. We found that the DQC echo intensity is largest at the center of the resonator bandwidth.

Table S3. DEER acquisition parameters, modulation depths, and SNR.

	15R1/28R1		21R1/38R1
GB1 Concentration (μM)	10	0.05	10
$(\pi/2)\nu_A$ (ns)	6	6	6
$(\pi)\nu_A$ (ns)	12	12	12
$(\pi)\nu_B$ (ns)	8	12	8
τ (ns)	400	400	400
T (ns)	2600	2400	3000
Δt (ns)	100	100	100
d30 (ns)	12	12	12
Frequency offset (MHz)	70	70	70
d31, J	4, 8	4, 8	4, 8
a	200	200	200
Shots per point	20	20	20
Scans	1	2	2
SRT (ms)	3	4	1
Attenuation (dB)	3	3	3
Points	199	182	224
Phase cycling	16-step	16-step	16-step
Modulation depth (%)	43	20	36
SNR (λ/σ_N)	86	10	199
Run time (hours)	1.5	12	1

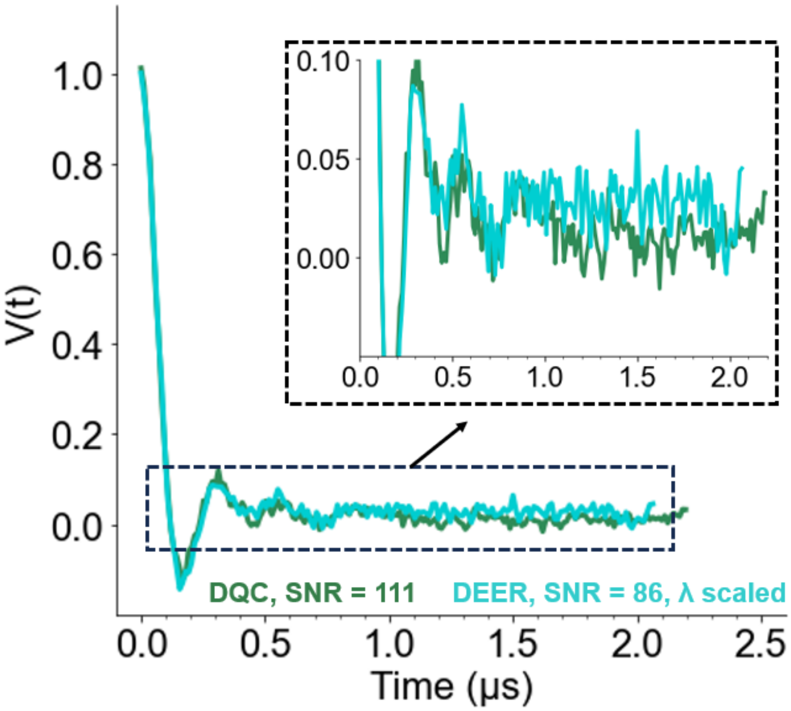

Figure S5. Background-subtracted DQC (green) and DEER (blue) time traces of 10 μM 15R1/28R1 GB1. The DEER time trace has been scaled to the 100% modulation depth of the DQC time trace. The inset shows a zoomed-in portion of the spectrum to highlight the noise amplitudes.

Analysis of pulsed dipolar ESR time traces

There are multiple analysis programs for pulsed dipolar experiments, however, the analysis of DQC data is not well-documented with these programs. We tested the most common software with the 10 μM GB1 mixture DQC data (Figure 4 in Main Text). We also tested the software on the 50 nM 15R1/28R1 GB1 DQC data (Figure 5 in Main Text). Analysis comparisons are shown in Figures S6 and S7.

We performed Tikhonov regularization in DeerAnalysis[8]. Due to the low background signal of the DQC time traces, we were unable to perform any background correction of the data using DeerAnalysis. Distance distributions were able to be estimated without background correction using Tikhonov regularization with the L-curve method. However, because there was no background correction, it was impossible to validate the obtained distance distributions without additional user input. In the validation window, we added white noise to the data with a level of 1.5 for each data set and set the background from 530 ns to 700 ns. We ran 110 trials of validation using this method. L-curves for all processed data are provided in Figures S9 and S11.

Additionally, neural network analysis was performed on the primary experimental data using DEERNet[9,10]. The uncertainty in the distributions is calculated as a 95% confidence interval from variation between 24 neural networks. We also performed Tikhonov regularization in DeerLab[11]. We ran the analyses with the compactness penalty and with bootstrapping of 1000 samples. Error was calculated as 95% confidence intervals.

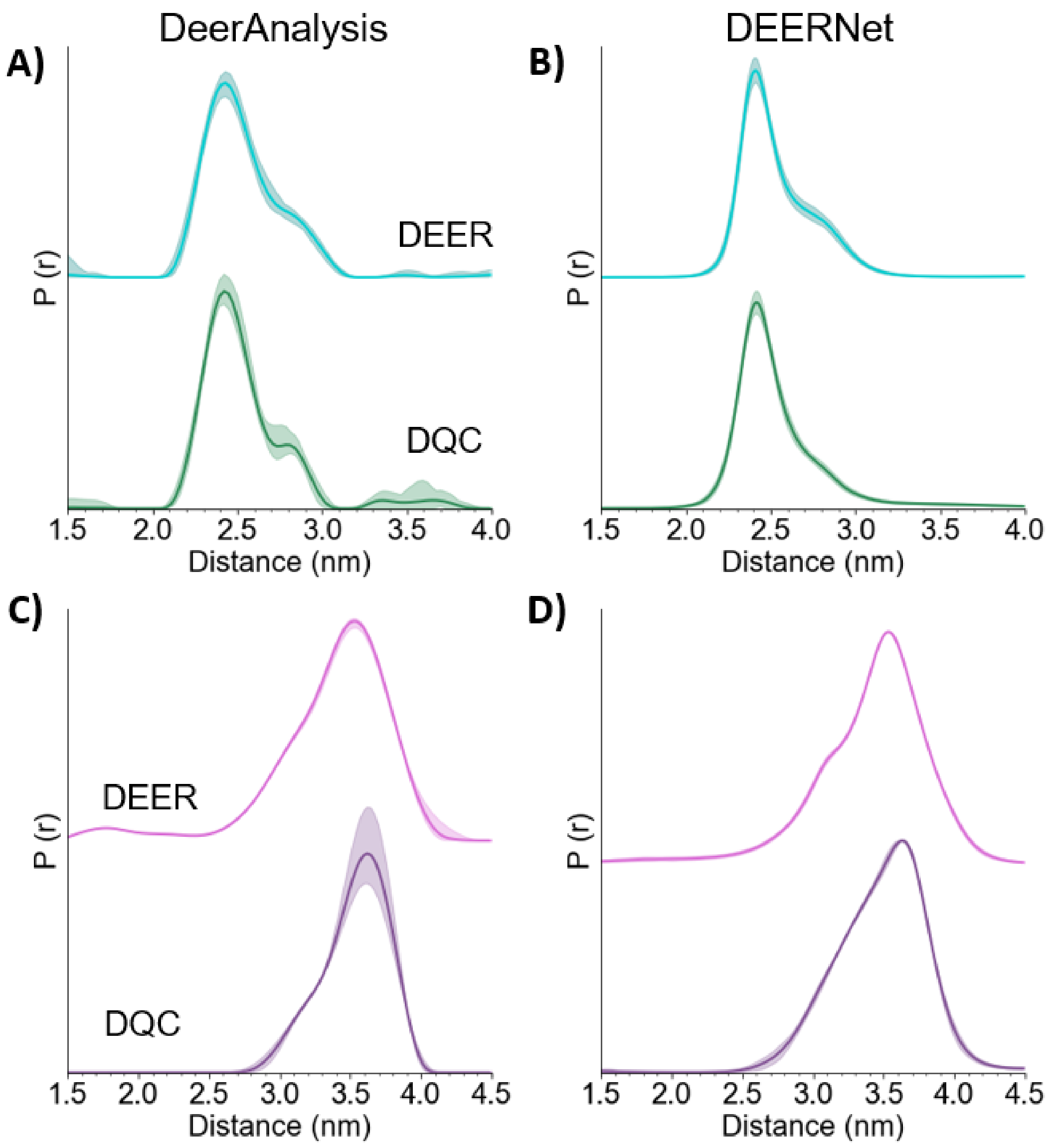

Figure S6. Comparison of analysis methods between DEER and DQC. 15R1/28R1 DEER (blue) and DQC (green) using (A) DeerAnalysis and (B) DEERNet. 21R1/38R1 DEER (pink) and DQC (purple) using (C) DeerAnalysis and (D) DEERNet.

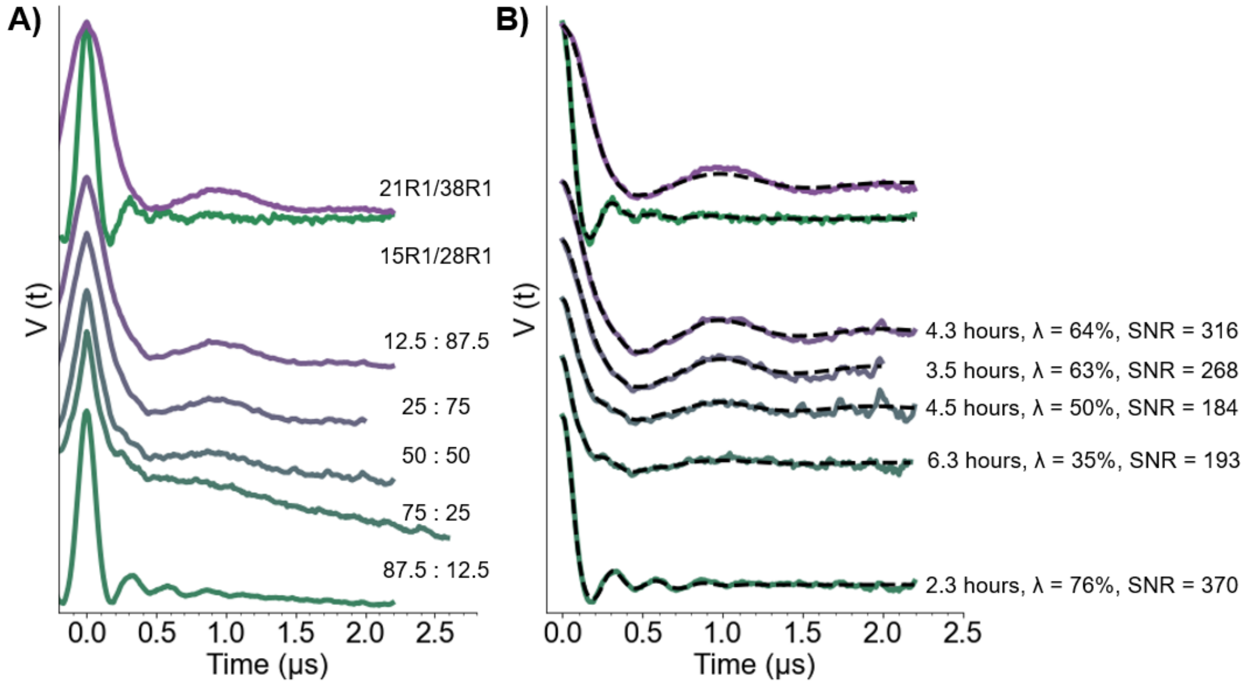

Figure S7. (A) Raw DQC time traces of GB1 mutants and mixtures of mutants. (B) Background-subtracted DQC time traces. Run time, modulation depths, and SNR are shown. The artifacts near the end of the time traces for the 50:50 and 75:25 mixtures are likely due to deuterium nuclear modulation effects that were not fully suppressed.

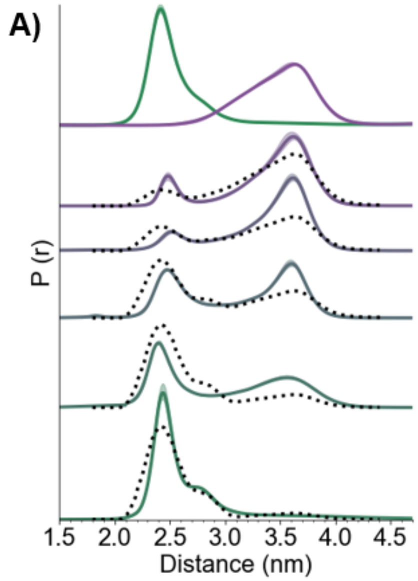

Figure S8. Comparisons of analysis methods for 10 uM 15R1/28R1 DQC. (A) DEERNet, (B) DeerAnalysis, (C) DeerLab with Bootstrapping. Dotted black lines represent the addition of the two distance distributions for the 15R1/28R1 and 21R1/38R1 GB1 mutants. From top to bottom: 15R1/28R1 GB1 (green) and 21R1/38R1 GB1 (purple), 12.5:87.5 mixture of 15R1/28R1 to 21R1/38R1, 25:75 mixture, 50:50 mixture, 75:25 mixture, 87.5:12.5 mixture.

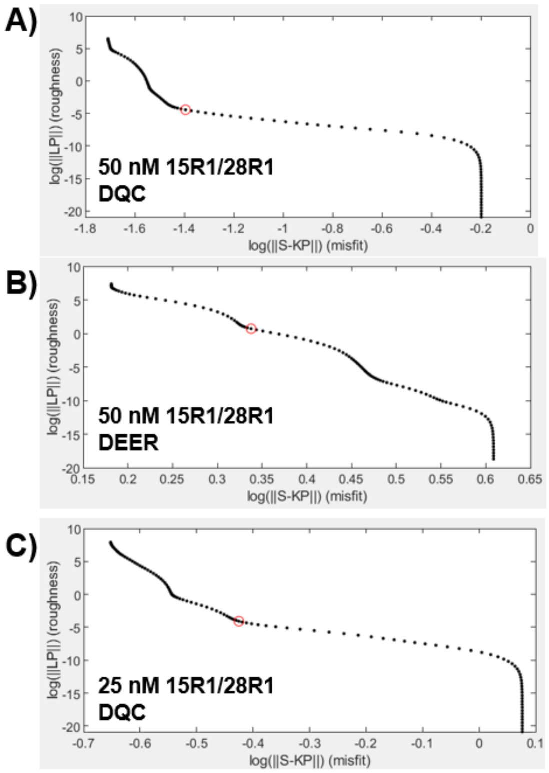

Figure S9. L-curve estimations of the Tikhonov regularization parameter using DeerAnalysis for (A) 50 nM 15R1/28R1 GB1 DQC, (B) 50 nM 15R1/28R1 GB1 DEER, and (C) 25 nM 15R1/28R1 GB1 DQC. The L-curve for the 50 nM DEER sample is not ideal, making the distribution difficult to interpret.

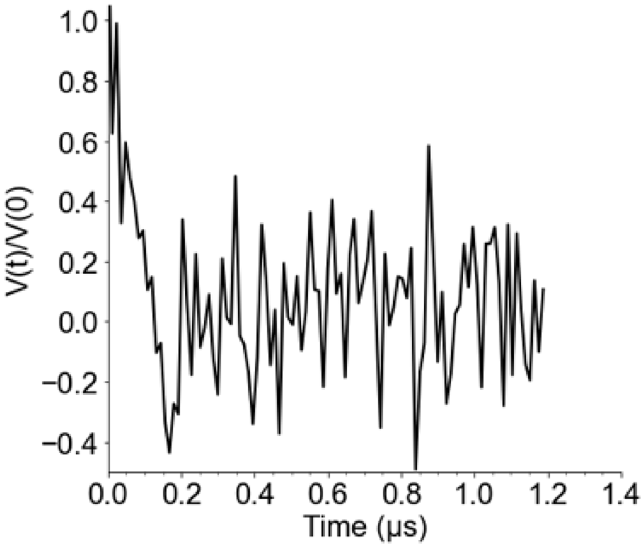

Figure S10. 10 nM 15R1/28R1 GB1 DQC. 50 K for 36 hours. Parameters are provided in Table S2.

Table S4. Comparison of DEER experiments performed with different equipment and parameters.

	Ackermann, et. al.[12]	*This work*
Resonator	ER 5106QT-2w<br>Cavity	Bridge12 QLP<br>Loop-Gap
*Resonator active volume (µL)**	52	4
DEER observer π pulse length (ns)	32	12
TWT amplifier (W)	150	300
*Protein Concentration (*µM)	25	10
Modulation depth (%)	35	43
SNR	92**<br>70 minutes	86<br>90 minutes

**calculated as the volume of a cylinder using the inner diameter of sample tube and resonator heights*
***estimated from Supplementary Information*[12]

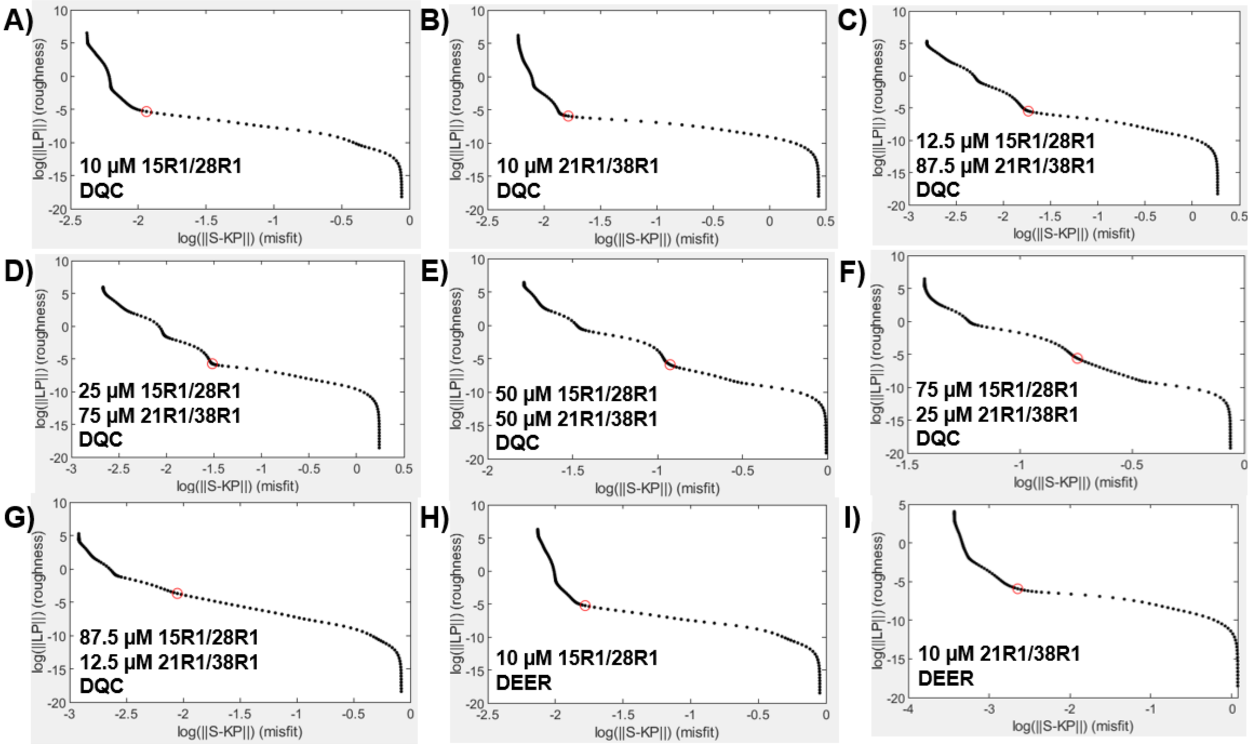

Figure S11. L-curve estimations of the Tikhonov regularization parameter using DeerAnalysis for (A) 10 µM 15R1/28R1 GB1 DQC, (B) 10 µM 21R1/38R1 GB1 DQC, (C)-(G) Mixtures of 15R1/28R1 GB1 and 21R1/38R1 GB1 DQC. (H) 10 µM 15R1/28R1 GB1 DEER, and (I) 10 µM 21R1/38R1 GB1 DEER.

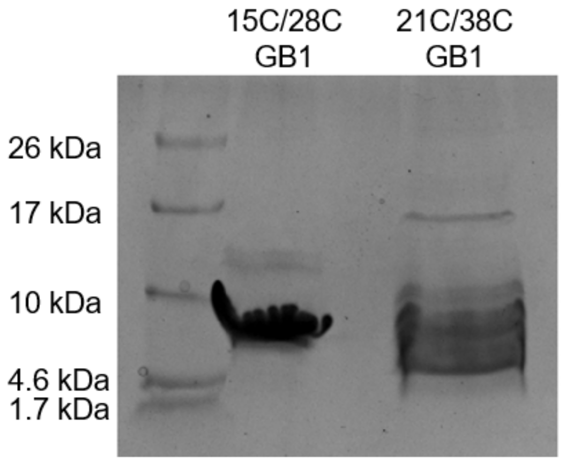

Figure S12. Purification of the GB1 mutants. 20% SDS-PAGE gels containing purified GB1 mutants. Lane 1 is the Spectra Multicolor Low Range Protein Ladder (Thermo Fisher Scientific). Lanes 2 and 3 are the GB1 mutants. The 56-residue GB1 has a molecular weight of approximately 8 kDa. The bands in the gel between the 4.6 and 8 kDa ladder bands indicate the presence of GB1.

REFERENCES

(1) Cunningham, T. F.; McGoff, M. S.; Sengupta, I.; Jaroniec, C. P.; Horne, W. S.; Saxena, S. High-Resolution Structure of a Protein Spin-Label in a Solvent-Exposed β-Sheet and Comparison with DEER Spectroscopy. *Biochemistry* **2012**, *51* (32), 6350–6359. https://doi.org/10.1021/bi300328w.

(2) Cunningham, T. F.; Pornsuwan, S.; Horne, W. S.; Saxena, S. Rotameric Preferences of a Protein Spin Label at Edge-Strand β-Sheet Sites: R1 Rotameric Preferences in β-Sheet Edge Strands. *Protein Science* **2016**, *25* (5), 1049–1060. https://doi.org/10.1002/pro.2918.

(3) Singewald, K.; Wilkinson, J.; Saxena, S. Copper Based Site-Directed Spin Labeling of Proteins for Use in Pulsed and Continuous Wave EPR Spectroscopy. *BIO-PROTOCOL* **2021**, *11* (24). https://doi.org/10.21769/BioProtoc.4258.

(4) Pannier, M.; Veit, S.; Godt, A.; Jeschke, G.; Spiess, H. W. Dead-Time Free Measurement of Dipole-Dipole Interactions between Electron Spins. *J Magn Reson* **2000**, *142* (2), 331–340. https://doi.org/10.1006/jmre.1999.1944.

(5) Borbat, P. P.; Freed, J. H. Multiple-Quantum ESR and Distance Measurements. *Chemical Physics Letters* **1999**, *313* (1–2), 145–154. https://doi.org/10.1016/S0009-2614(99)00972-0.

(6) Saxena, S.; Freed, J. H. Theory of Double Quantum Two-Dimensional Electron Spin Resonance with Application to Distance Measurements. *The Journal of Chemical Physics* **1997**, *107* (5), 1317–1340. https://doi.org/10.1063/1.474490.

(7) Borbat, P. P.; Freed, J. H. Double-Quantum ESR and Distance Measurements. In *Biological Magnetic Resonance*; Academic/Plenum Publishers: New York, 2000; Vol. 19, p 385.

(8) Jeschke, G.; Chechik, V.; Ionita, P.; Godt, A.; Zimmermann, H.; Banham, J.; Timmel, C. R.; Hilger, D.; Jung, H. DeerAnalysis2006—a Comprehensive Software Package for Analyzing Pulsed ELDOR Data. *Appl. Magn. Reson.* **2006**, *30* (3–4), 473–498. https://doi.org/10.1007/BF03166213.

(9) Worswick, S. G.; Spencer, J. A.; Jeschke, G.; Kuprov, I. Deep Neural Network Processing of DEER Data. *Sci. Adv.* **2018**, *4* (8), eaat5218. https://doi.org/10.1126/sciadv.aat5218.

(10) Keeley, J.; Choudhury, T.; Galazzo, L.; Bordignon, E.; Feintuch, A.; Goldfarb, D.; Russell, H.; Taylor, M. J.; Lovett, J. E.; Eggeling, A.; Fábregas Ibáñez, L.; Keller, K.; Yulikov, M.; Jeschke, G.; Kuprov, I. Neural Networks in Pulsed Dipolar Spectroscopy: A Practical Guide. *Journal of Magnetic Resonance* **2022**, *338*, 107186. https://doi.org/10.1016/j.jmr.2022.107186.

(11) Fábregas Ibáñez, L.; Jeschke, G.; Stoll, S. DeerLab: A Comprehensive Software Package for Analyzing Dipolar Electron Paramagnetic Resonance Spectroscopy Data. *Magn. Reson.* **2020**, *1* (2), 209–224. https://doi.org/10.5194/mr-1-209-2020.

(12) Ackermann, K.; Wort, J. L.; Bode, B. E. Nanomolar Pulse Dipolar EPR Spectroscopy in Proteins: Cu^{II}–Cu^{II} and Nitroxide–Nitroxide Cases. *J. Phys. Chem. B* **2021**, *125* (20), 5358–5364. https://doi.org/10.1021/acs.jpcb.1c03666.
